# Feeling Music: Integration of Auditory and Tactile Inputs in Musical Meter Perception

**DOI:** 10.1371/journal.pone.0048496

**Published:** 2012-10-31

**Authors:** Juan Huang, Darik Gamble, Kristine Sarnlertsophon, Xiaoqin Wang, Steven Hsiao

**Affiliations:** 1 Zanvyl Krieger Mind/Brain Institute and the Solomon H. Snyder Department of Neuroscience, The Johns Hopkins University, Baltimore, Maryland, United States of America; 2 Laboratory of Auditory Neurophysiology, Department of Biomedical Engineering, The Johns Hopkins University, Baltimore, Maryland, United States of America; McMaster University, Canada

## Abstract

Musicians often say that they not only hear, but also “feel” music. To explore the contribution of tactile information in “feeling” musical rhythm, we investigated the degree that auditory and tactile inputs are integrated in humans performing a musical meter recognition task. Subjects discriminated between two types of sequences, ‘duple’ (march-like rhythms) and ‘triple’ (waltz-like rhythms) presented in three conditions: 1) Unimodal inputs (auditory or tactile alone), 2) Various combinations of bimodal inputs, where sequences were distributed between the auditory and tactile channels such that a single channel did not produce coherent meter percepts, and 3) Simultaneously presented bimodal inputs where the two channels contained congruent or incongruent meter cues. We first show that meter is perceived similarly well (70%–85%) when tactile or auditory cues are presented alone. We next show in the bimodal experiments that auditory and tactile cues are integrated to produce coherent meter percepts. Performance is high (70%–90%) when all of the metrically important notes are assigned to one channel and is reduced to 60% when half of these notes are assigned to one channel. When the important notes are presented simultaneously to both channels, congruent cues enhance meter recognition (90%). Performance drops dramatically when subjects were presented with incongruent auditory cues (10%), as opposed to incongruent tactile cues (60%), demonstrating that auditory input dominates meter perception. We believe that these results are the first demonstration of cross-modal sensory grouping between any two senses.

## Introduction

When listening to music in a concert hall or through loud speakers or when playing music on an instrument, we not only hear music but also have the experience of “feeling” the music in our bodies. The neural basis of what it means to “feel” music is not understood, however the term “feeling” suggests that it may involve other sensory inputs besides audition, such as inputs from proprioceptive, vestibular, and/or tactile cutaneous afferents from the somatosensory system. In this study we explored whether inputs from cutaneous afferents, which heavily innervate the skin and deep tissues of the body, contribute to meter perception. In addition to pitch and timbre, music is distinguished by the delicate temporal processing of the sequence of notes that give rise to rhythm, tempo, and meter, which is the focus of this study. Meter is defined as the abstract temporal structure that corresponds to periodic regularities of music [1], [2]. It is based on the perception of regular beats that are equally spaced in time [3], [4]. Meter is perceived as “duple” (or march-like) when a musical measure (the primary cycle of a set of notes) is subdivided into two or four beats, and “triple” (or waltz-like) when subdivided into three beats [5]. Whether a piece of music is perceived as duple or triple often depends on the emphasis (increased duration, change in frequency, or amplitude) placed on the first beat (downbeat) of a measure [6]. Meter is also strongly influenced by the probability of when the accent cues occur at key metrically positioned notes within a measure [2], [4], [7]. The frequency of occurrence of salient events or accents at these metrically important positions is an important [5] but not critical cue to meter perception since meter can be perceived even if all of the sounds in a regular sequence are physically identical [8], [9]. In this case, the only cue that can be used to extract meter information is the probability of the occurrence of notes at the metrically important positions in the temporal sequence.

While auditory cues are clearly important for signaling meter, it has been shown that meter perception can be influenced by inputs from other sensory modalities. For example, people tend to tap, dance, or drum to the strong beats of a musical rhythm, demonstrating the close relationship between movement and rhythm [10], [11]. Of particular relevance here are studies by Brochard et al. [11] who showed that meter can be perceived by tactile inputs. In their study, they showed that subjects can tap to the meter of a musical piece with their right hand when presented with corresponding mechanical tactile pulses to their left hand. In other studies, Trainor and her colleagues [12]–[16] showed that inputs to the vestibular system during the metrically important notes can be used to disambiguate whether a tone sequence is duple or triple supporting the notion that head movements can also influence meter perception. It is doubtful that the visual system plays a role in meter perception since studies show that meter cannot be extracted from purely visual input [15], [17], and recent studies show that visual rhythm perception is much poorer in vision than audition [44]. Taken together, previous studies suggest that musical meter perception is a multi-modal process that integrates vestibular, somatosensory and auditory inputs. The question remains of how inputs from the other modalities interact with auditory inputs when perceiving meter. In this study we addressed this question and investigated whether meter perception is an example of perceptual cross modal grouping which has not been demonstrated previously across any senses [43].

To test the role of touch in meter perception, we conducted four meter recognition tests on subjects with normal hearing using ‘duple-tending’ and ‘triple-tending’ note sequences presented separately and together to the auditory and tactile systems in different combinations. In experiment 1 we show that meter can be extracted from either unimodal auditory or tactile sequences. In experiments 2 and 3 we show that cross-modal grouping of inputs from touch and audition occurs in meter perception. In experiment 4 we show that meter is a single percept and that when given both tactile and auditory inputs simultaneously, auditory cues dominate.

## Materials and Methods

### Participants

Twelve healthy musically trained participants (9 female; mean age = 19.3±1.6 years; years of playing musical instruments = 7.75±3.5) took part in the experiments. Two subjects did not complete all of the testing sessions. All participants reported that they had normal hearing and tactile sensation. They were first tested for their ability to perceive meter using the Montreal Battery of Evaluation of Amusia (MBEA) [41] with all of the subjects performing above 94% correct. All of the subjects were naïve to the purpose of the study and to the test procedures and gave their written informed consent for participating in the experiments. All of the testing procedures were approved of by the human institutional review board of the Johns Hopkins University. No minors participated in the study.

### Experiment Setup

Auditory stimuli were delivered to the left ear of participants from circumaural sealed headphones (HDA 200, Sennheiser, Old Lyme, CT) via a Crown D-75A amplifier (Crown Audio and IOC Inc., Elkhart, IN). Tactile stimuli were delivered along the axis perpendicular to the left index finger of participants by a circular contact (8 mm diameter) connected to a Chubbuck motor [42]. The motor was mounted to an adjustable stage (UMR8.51, Newport Corp., Irvine, CA) that was supported by a custom-built aluminum frame, which was placed within in a sound-attenuation chamber. The stimulator noise generated by the Chubbuck motor was inaudible even without wearing the headphones. The participant placed his or her hand through an entry hole (lined with foam) and rested their hands on a support platform in a supinated position mounted directly below the contact probe. The probe was lowered (via the adjustable stage actuator) until it firmly contacted the skin (about 1 mm indentation). The Chubbuck motor is equipped with a high precision LVDT with micron-resolution. The output of the LVDT and the Crown D-75A were digitized (PCI-6229, National Instruments, Austin, TX; sampling rate = 5 kHz). During all of the experiments, subjects always wore the headphones and kept their left index finger in contact with the probe. Participants adjusted the amplifiers to set both the auditory and tactile stimulation at a level that felt comfortable.

### Stimulus

Stimuli were sequences consisting of 24 notes that were each 500 ms in duration. Fifteen of the temporal units were notes and nine were silent. Each note consisted of a 350 ms sinusoidal tone (220 Hz (A3)) or a vibration (220 Hz) followed by a 150 ms silent period. The onset and offset of each of the notes were ramped on and off within a 35 ms time window. Silent units consisted of 500 ms of silence. Each note simulated a beat in a musical sequence, which were equally spaced points in time, either in the form of sounded events (the 15 notes which were tones that were played to the ear or vibrations that were played to the skin) or silent events (the 9 notes where no stimulation was delivered).

Given the constraint of 15 stimulated and 9 silent notes, all possible 24-unit sequences were generated. Sequences were retained only if: 1) The first and last units in every sequence contained a note; 2) The sequence did not contain three or more successive silent units; 3) The number of notes occurring in every odd unit was less than 12. These sequences were classified into ‘duple-tending’ and ‘triple-tending’ in a manner adapted from previous studies [7], [8].

**Figure 1:**
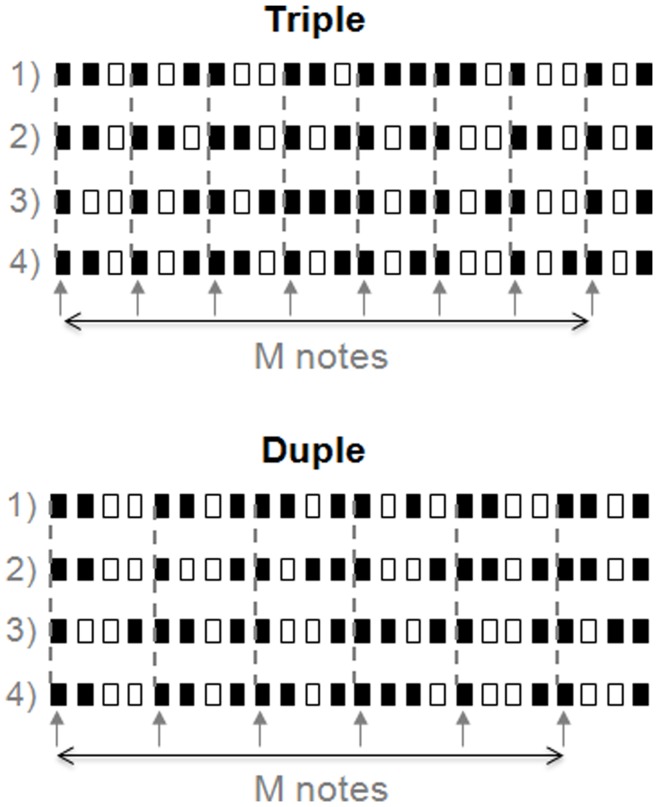
Examples of triple and duple sequences. Stimuli are event sequences with different rhythms composed of 24 temporal units that were 500 ms in duration, nine of which were silent units (open bars) and 15 of which were note units (dark bars). Each open bar represents a 500 ms silence. Each dark bar represents a note (pure tone/sinusoidal vibration) with a duration of 350 ms followed by 150 ms of silence. Triple sequences consisted of 8 notes (every third unit) in metrically important positions (M notes) and 7 notes in metrically unimportant positions (N notes). Duple sequences consisted of 6 M notes (every fourth unit) and 9 N notes. Arrows and dashed lines indicate M notes in the sequences.

**Figure 2:**
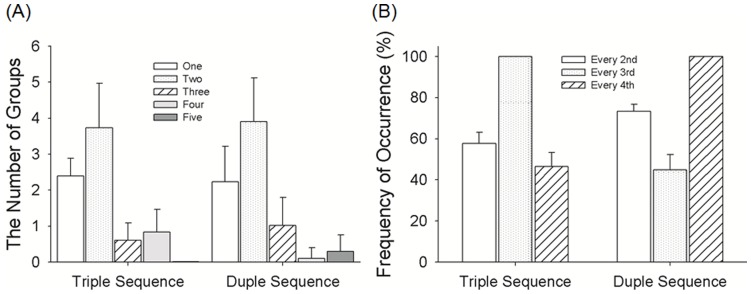
Statistics of the 374 triple and 331 duple sequences. Figure 2A shows the number of successive notes in groups of 1, 2, 3, 4, and 5 occurring in the pool of sequences, indicating the same statistics for Triple and Duple sequences. Figure 2B shows the frequency of a note occurring at every 2nd, 3rd, and 4th unit in the pool of sequences, no difference between Triple and Duple sequences.

In ‘duple-tending’ sequences: 1) the number of notes occurring was 6 in every fourth unit (100% of metrically important position) and fewer than 3 of which could have notes in the units immediately before and after it; 2) fewer than 4 notes occur in every third unit. In ‘triple-tending’ sequences: 1) the number of notes occurring in every third unit was 8 (100% of metrically important position) and fewer than 3 of which could have notes in the units immediately before and after it; 2) fewer than 3 notes occur in every fourth unit. Therefore, ‘triple-tending’ sequences had a temporal structure consisting of a group of three ‘beats’, and ‘duple-tending’ sequences had a temporal structure consisting of a group of ‘four beats’. In total, there were 374 triple and 331 duple sequences. Four examples of triple and duple sequences are shown in Figure 1. Statistics of the 374 triple and 331 duple sequences are shown in Figure 2A. Figure 2B shows the frequency of note occurrences. Notes were classified as metrically important (M notes) or metrically unimportant (N notes) depending on where they were located in the temporal sequence (Fig. 1). Triple sequences contained 8 M notes and 7 N notes. Duple sequences contained of 6 M notes and 9 N notes. Stimuli were generated digitally and converted to analog format (PCI-6229, National Instruments, Austin, TX; sampling rate = 44.1 kHz) and delivered to either the headphone or to the Chubbuck tactile stimulator [42].

### Procedures

**Figure 3:**
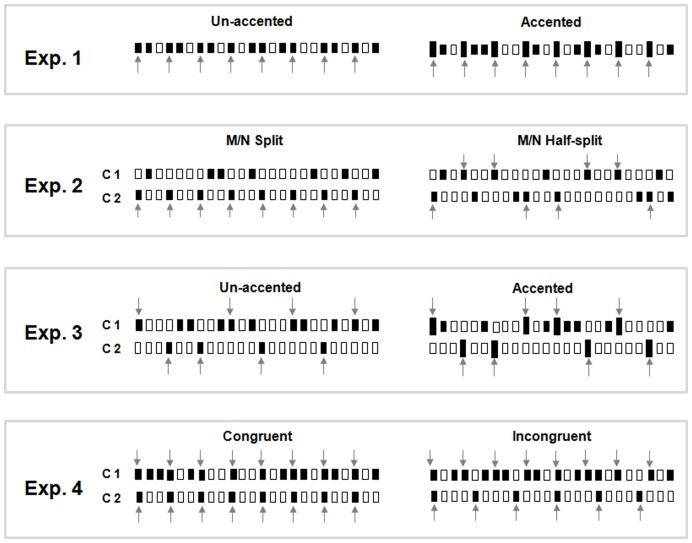
Test trial of Triple sequence examples. Each open bar represents a 500 ms silence. Each dark bar represents a note (pure tone/sinusoidal vibration) with a duration of 350 ms followed by 150 ms of silence. Larger dark in accented trials represent an amplitude accent, with 20 dB higher amplitude than regular dark notes. Exp. 1: unimodal trial, a whole triple sequence is assigned to either auditory or tactile modality. Ex. 2 and 3: bimodal trials, dark bars in channel 1 and channel 2 together compose a whole triple sequence. Exp. 4: bimodal trials, channel 1 contain a whole triple sequence, channel 2 contain additional Triple (Congruent) or Duple (Incongruent) M notes. For Exp. 2, 3, and 4, channel 1 notes sent to one modality and channel 2 notes sent to another modality. Channel 1 and 2 notes are assigned to either auditory and tactile modalities, or tactile and auditory modalities, respectively.

Four meter-recognition experiments were conducted (Fig. 3). Table 1 lists the conditions that were tested in each experiment. Triple and duple tending sequences consisted of 24 trials respectively for each of the conditions listed in Table 1. Auditory stimulation was presented through a headphone to the subject’s right ear and tactile stimulation presented to the subject’s left index fingertip with probe attached to a Chubbuck tactile stimulator (see above). Before beginning the experiments, subjects were given a practice session and were instructed to listen to strongly cued duple and triple meter sequences and to respond on a custom-written computer interface whether the sequence they had just heard was duple (presented in groups of four) or triple (presented in groups of three). Subjects were told whether a unimodal or bimodal block was being played, but were never directed to attend specifically to either modality. Subjects received no feedback on their responses.

**Table 1:** Test conditions for each experiment.

Test	Test condition
**Exp 1**	**Un-accented**
	A. whole sequence to Aud. only
	B. whole sequence to Tac. only
	**Accented**
	C. whole sequence with accented M notes to Aud. only
	D. whole sequence with accented M notes to Tac. only
**Exp 2**	**M/N Split**
	A. Aud C1/Tac C2
	a) Bimodal: N notes to Aud and M notes to Tac.
	b) Unimodal: N notes to Aud. only
	**M/N Half-split**
	A. Aud C1/Tac C2
	a) Bimodal: ½ M notes and ½ N notes to Aud. and the rest of the notes to Tac
	b) Unimodal: ½ M notes and ½ N notes to Aud. only
	B. Tac C1/Aud C2
	a) Bimodal: ½ M notes and ½ N notes to Tac. and the rest of the notes to Aud.
	b) Unimodal: ½ M notes and ½ N notes to Tac. only
**Exp 3**	**Un-accented**
	A. Aud C1/Tac C2
	a) Bimodal: ½ M notes and all N notes to Aud and ½ M notes to Tac.
	b) Unimodal: ½ M notes and all N notes to Aud only
	B. Tac C1/Aud C2
	a) Bimodal: ½ M notes and all N notes to Tac and ½ M notes to Aud.
	b) Unimodal: ½ M notes and all N notes to Tac only
	**Accented**
	A. Aud C1/Tac C2
	a) Bimodal: ½ accented M notes and all N notes to Aud and ½ accented M notes to Tac.
	b) Unimomdal: ½ accented M notes and all N notes to Aud only
	B. Tac C1/Aud C2
	a) Bimodal: ½ accented M notes and all N notes to Tac and ½ accented M notes to Aud.
	b) Unimodal: ½ accented M notes and all N notes to Tac only
**Exp. 4**	**Congruent**
	A. Aud C1/Tac C2
	a) Whole triple sequence to Aud. and triple M notes to Tac.
	b) Whole duple sequence to Aud. and duple M notes to Tac.
	B. Tac C1/Aud C2
	a) Whole triple sequence to Tac. and triple M notes to Aud.
	b) Whole duple sequence to Tac. and duple M notes to Aud.
	**Incongruent**
	A. Aud C1/Tac C2
	a) Whole triple sequence to Aud. and duple M notes to Tac.
	b) Whole duple sequence to Aud. and triple M notes to Tac.
	B. Tac C1/Aud C2
	a) Whole triple sequence to Tac. and duple M notes to Aud.
	b) Whole duple sequence to Tac. and triple M notes to Aud.

A total of 24 test conditions were presented pseudo-randomly to the subjects in 6 test sessions, with 2 for unimodal and 4 for bimodal tests. Each test block contained an equal number of triple and duple test sequences drawn randomly from the pool of 374 triple and 331 duple sequences. Each test block consisted of 24 trials. Trials within each block were presented in a randomized order, and test blocks were also repeated in a randomized order. Each subject was tested with 5–6 blocks in 1-hour test sessions. Subjects were allowed to take a break every two blocks. Six test sessions were scheduled for each subject with one session per day. All of the subjects completed the study within 6 weeks.

### Data Analysis

Subject’s responses were automatically recorded and a response was scored as correct if the subject’s response matched the assigned meter of the sequence. In the incongruent condition in experiment 4 a response was considered correct if it matched the meter assigned to channel1 (C1). The percentage of correct responses for each condition was calculated with triple and duple sequences analyzed separately. We carried out a *d’* analysis for experiments 1, 2 and 3. Group sensitivity *d’* values based on the mean hit- and false-alarm rates of subjects were compared between unimodal and bimodal testing conditions. Hit rate was defined as a triple response when the stimulus was a triple sequence, and false-alarm rate as a triple response when the stimulus was a duple sequence. *d’ = *z(P_triple_) – z(1 - P_duple_).

In the next section we describe the specific details and results of the four experiments separately.

## Results

### Experiment 1: Meter Perception via Unimodal Stimulation

#### Test condition

**Figure 4:**
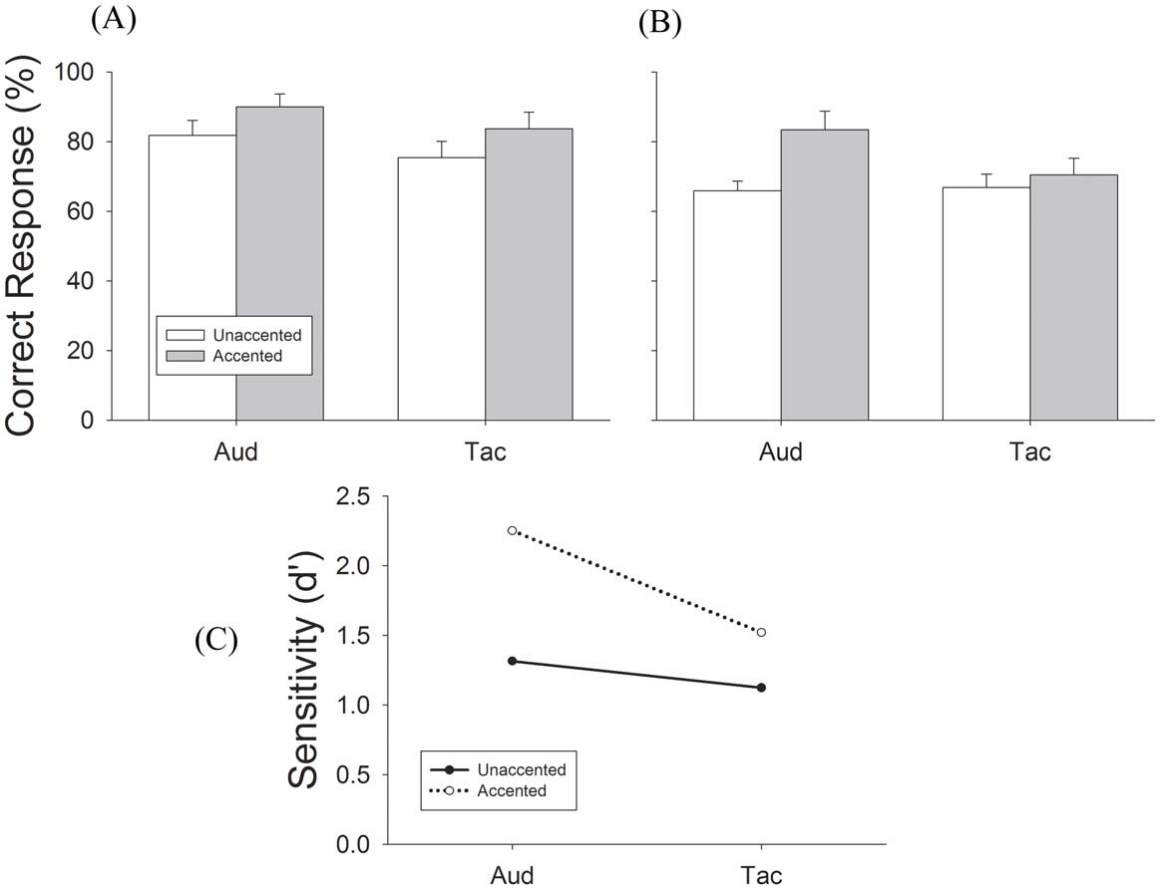
Results of Experiment 1, unimodal meter perception performance. (A) Performance for Triple-tending sequence. (B) Performance for Triple-tending sequence. Open bars represent unaccented condition, gray bars represent accented conditions. Error bars are Standard Error. (C) Solid line is unaccented metric note trials and dashed line is accented metric note trials.

In Experiment 1 we examined the ability of subjects to perceive meter with unimodal presentation (auditory or tactile input alone) of the note sequences (Figure 2). Subjects were asked to perform two tasks (Table 1): (1) Un-accented task where all of the notes had the same intensity, and (2) Accented task where an amplitude accent (+20 dB) was applied to the metrically important (M) notes (Figure 3). Experiment 1 has four test conditions (Table 1). Each test condition was tested in 16 trials for triple- and duple-tending sequences, respectively, with a total of 128 trials. Results of Experiment 1 are shown in Figure 4.

#### Results of experiment 1

The mean correct responses for unaccented triple sequences through auditory and tactile stimuli alone was 82% and 75%, respectively (Fig. 4A). Adding amplitude cues increased performance to 90% and 84% for auditory and tactile stimuli, respectively (Fig. 4A). Results for duple perception showed the same trend (Fig. 4B). Accented M notes significantly enhanced meter perception performance for both auditory and tactile unimodal test conditions, with a larger enhancement for the auditory condition (Fig. 4C). The comparison of d’ values between auditory and tactile conditions suggests that accented cues may be weighted more heavily in audition than in touch when perceiving meter (Fig. 4C).

We performed a repeated measure two-way (meter and accent) ANOVA to examine the difference between unimodal auditory and tactile meter perception. A significant difference was found in triple [F_(1,12)_ = 10.89, p = .006] but not in duple [F_(1,12)_ = 2.71, p = .13] perception. Paired *t-test* results show that triple meter perception through auditory stimulation is significantly stronger than tactile stimulation in the accented condition [T = 2.96, p = .01] but not in the unaccented condition [T = 2.02, p = .07] (Fig. 4A). Further, a significant main effect of accent was observed in both triple [F_(1,12)_ = 8.94, p = .01] and duple [F_(1,12)_ = 10.84, p = .006] sequences. Accenting the M notes significantly increased performance for triple sequences in both auditory [T = 2.69, p = .02] and tactile conditions [T = 2.29, p = .04]. The enhancement was also seen in duple sequences in auditory [T = 3.18, p = .008], but not tactile conditions [T = 1.13, p = .28]. These results agree with previous studies of auditory meter perception [2], [4]. We conclude from experiment 1 that meter perception through touch is similar to meter perception through audition with touch being less sensitive to amplitude cues (see discussion).

### Experiment 2: Bimodal Grouping of Meter Perception between Touch and Audition

#### Test condition

**Figure 5:**
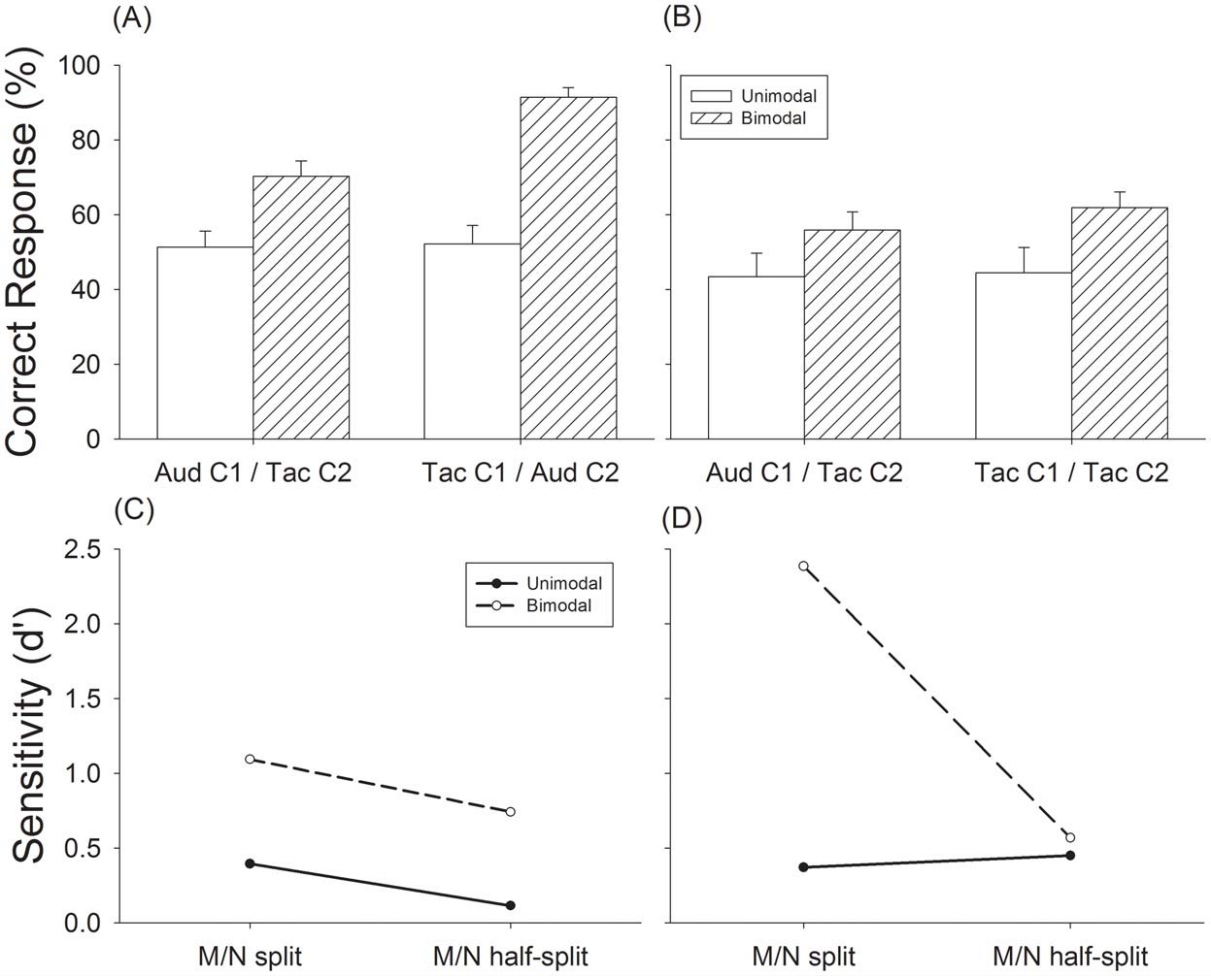
Results of Experiment 2, meter recognition performance in triple sequences for bimodal M/N split and M/N half split tasks. (A) triple sequences tested in the M/N split task, (B) triple sequences tested in the M/N half-split task, (C) discriminability analysis of meter perception in the M/N split and M/N half-split tasks with Aud-C1/Tac-C2 condition, (D) discriminability analysis of meter perception in the M/N split and M/N half-split tasks with Tac-C1/Aud-C2 conditions. Open bars are results tested under unimodal condition. Hashed bars are results tested under bimodal condition. Error bars are standard error.

In Experiment 2 (Fig. 3, Exp. 2, Table 1), we examined the degree that inputs from the auditory and tactile channels are grouped in meter perception. In these experiments, a sequence was disassembled and notes were assigned partly to channel and partly to the other channel. The change in meter perception performance under these conditions with respect to unimodal conditions was used to indicate whether auditory-tactile grouping occurs in meter perception. In Experiment 2, the M and N notes were distributed between the two modalities in two ways. In the first, called “M/N split”, the N notes were presented to one channel and the M notes were presented to the other channel. In the second, called “M/N half-split” task, half of the M notes and half of the N notes were randomly presented to the two channels (Fig. 3, Exp. 2). Experiment 2 has eight test conditions as listed in Table 1. Each test condition was tested in 16 trials for triple- and duple-tending sequences, respectively, resulting in a total of 256 test trials. As a control for the M/N split task, just the N notes were delivered to the auditory or tactile channels under unimodal conditions. Similarly, as a control for the M/N Half-split task, half of M notes and half of N notes were delivered to the auditory or tactile channels unimodal conditions (Table 1). Data from these unimodal control conditions resulted in subjects performing at chance and demonstrated that meter is poorly perceived within a single channel in either task or modality when subjects are only given half of the notes (Figure 5).

#### Results of experiment 2

In Experiment 2, we investigated whether meter perception is possible when the 15 M notes in a stimulus sequence were distributed between the two channels in a way that neither channel alone could produce a coherent meter percept (Figure 5). In the M/N split task, there was little, if any, metric information within a single channel (unimodal conditions) with performance at chance level (50%) for triple sequences (Fig. 5A) and performance slightly above chance (60%) for duple sequences (Fig. S1A). The enhanced performance supports previous findings that there is a “duple” bias when perceiving meter [16]. When the sequences were presented bimodally, subjects were able to perceive meter clearly for both triple meter (Fig. 5A) and duple meter (Fig. S1A). For triple sequences, the bimodal enhancement was 19% when the auditory channel contained the N notes and the tactile channel contained the M notes(i.e., channel 1 was auditory [F_(1,12)_ = 14.92, p = .002] and was further enhanced to 39% [F_(1,12)_ = 54.89, p<.001] when the tactile channel contained the N notes and the auditory channel contained the M notes (Fig. 5A). For duple sequences, bimodal enhancement was 7% [F_(1,12)_ = 2.05, p = .18] when the auditory channel contained the N notes and the tactile channel contained the M notes, and 22% [F_(1,12)_ = 22.87, p<.001] when the tactile channel contained the N notes and the auditory channel contained the M notes (Sup. Fig. 1A).

The bimodal meter perception performance in the M/N split task and Aud-C1/Tac-C2 condition (70.3%, Fig. 5A) was mildly poorer than the unimodal auditory condition shown in Experiment 1 (82%, Fig. 4A), but the performance in the bimodal M/N split task with the Tac-C1/Aud-C2 condition (92%, Fig. 5A) was better than the unimodal tactile condition (75%, Fig. 4A). The data from the M/N split task suggests that meter perception is grouped across touch and audition and that M notes play a larger role when presented through the auditory channel.

If inputs from touch and audition are grouped equally to form meter perception then it should not matter whether the M or N notes are assigned to either the auditory or tactile channel. In the second task (M/N half-split) we tested a less structured distribution of notes with the M and N notes evenly split between the two channels (Fig. 3 and Table 1). Again, subjects did not perceive triple meter in the unimodal conditions, with performance at 43% and 45% for the Aud-C1 and Tac-C1 conditions, respectively (Fig. 5B)-confirming that meter information was not present within a single channel under this condition. Again. a duple bias was present in these non-metric sequences with duple perception being greater than 60% and 70% for Aud-C1 and Tac-C1 conditions, respectively (Fig. S1B). In spite of this condition being more difficult than the M/N split condition in integrating the inputs, we observed that in the bimodal condition, the percent correct responses to triple sequences increased to 59% and 63% respectively (Fig. 5B). A paired t-test between the bimodal performance and chance was significant when the primary channel (C1) was auditory (t = 5.47, p<.01) or tactile (t = 4.88, p<.01). The enhancement of meter perception with bimodal presentation of the sequences was also observed when channel 1 was assigned to auditory but not tactile modality for duple stimuli (Fig. S1B).

A comparison of *d’* between unimodal and bimodal conditions showed that an incomplete note sequence presented from either modality does not give rise to meter perception, regardless of modality (Fig. 5C and Fig. 5D). Adding the remaining notes from the other channel produced reliable recognition of meter pattern to subjects especially in the M/N split task, where the *d’* value increased to 1.1 when channel 1 was audition (dashed line, Fig. 5C) and to 2.4 when channel 1 was touch (*dashed line*, Fig. 5D). When channel 1 was audition the d’ value increased in the M/N Half-split task as much as what we observed in the M/N Split task. Although the d’ values did not increase much in bimodal condition for M/N half-split task when channel 1 was tactile, the percentage of correct responses was significantly higher than chance level in both cases (Fig. 5B). These results show that subjects can recognize triple meter patterns in the bimodal conditions and provide evidence of cross-modal grouping of inputs presented separately to the auditory and tactile systems. A comparison of the d’ values between the M/N split and M/N half-split tasks showed that M and N notes randomly split and delivered to the two channels was less efficient than from a single channel in inducing meter perception (Fig. 5D). The results show that cross-modal grouping is more effective when important metric cues (M) are consistently played in one modality than when they are split between modalities.

### Experiment 3: Asymmetry between Auditory and Tactile Stimulation in Meter Perception

#### Test condition

In Experiment 3 we further tested the degree of bimodal grouping by assigning one channel all of the N notes and assigning half of the M notes to one channel and the other half to the other channel (Fig. 3, Exp. 3). In these experiments subjects received asymmetrical inputs to the two sensory inputs which we surmised could affect cross-modal grouping. Both “Un-accented” and “Accented” tasks were tested. An amplitude accent (+20 dB) was applied to the metrically important (M) notes in the “Accented” task. Experiment 3 had eight test conditions (Table 1). Each condition was tested in 16 trials for triple- and duple-tending sequences, respectively, resulting in a total of 256 test trials. As a control for both tasks, subjects were presented with unimodal input (Aud-C1 and Tac-C1) conditions containing half of the M and all of the N notes Data for unimodal control conditions confirmed that meter information was not present within a single channel in both tasks when subjects were given unimodal input.

#### Results for experiment 3

Experiment 1 showed that accented metric cues had a larger influence on auditory performance while Experiment 2 indicated that M notes presented from the auditory channel produced a stronger meter percept than when presented to the tactile channel. These results suggest that the influence of auditory and tactile inputs on meter perception is not symmetrical. In Experiment 3, we further explored this asymmetry. In this experiment, one channel contained all of the N notes and half of the M notes with the other channel containing the other half of the M notes (Fig. 3). This asymmetric distribution allowed us to observe modality dependent characteristics of meter perception. Meter is naturally extracted from unimodal input and as such, perception of meter from bimodal input should be affected differently by inputs from audition and touch:1) auditory input should less susceptible to influences from touch if C1 receives auditory input, and 2) that auditory inputs should strongly influence the perception of meter from touch.

**Figure 6:**
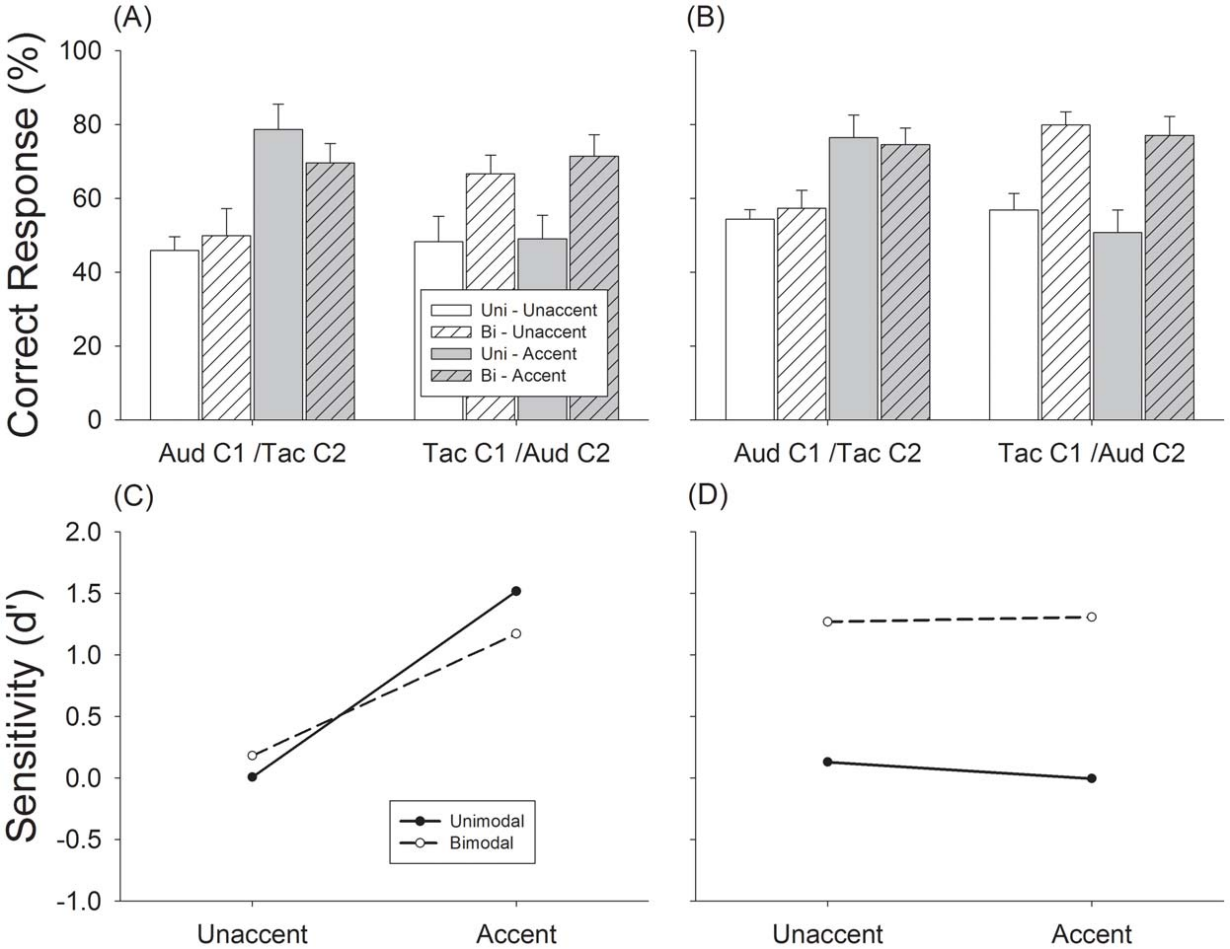
Results of Experiment 3, meter recognition performance with triple sequences (A) and duple sequences (B). (A) Correct responses to triple sequences. (B) Correct responses to duple sequences. Open bars: unaccented condition data. Gray bars: accented conditions. Hashed open bars: bimodal conditions. Hashed gray bars: bimodal accented conditions. Error bars are standard error. (C) Discriminability analysis of meter perception with Aud-C1/Tac-C2 condition; (D) Discriminability analysis of meter perception with Tac-C1/Aud-C2 condition. Open bars are unimodal unaccented control condition, hashed bars are bimodal unaccented condition; gray bars are unimodal accented control condition, and hashed gray bars are bimodal accented condition.

Again, un-accented unimodal control conditions produced chance-level performance for triple perception (open bars, Fig. 6A) and slightly above chance-level performance for duple perception (open bars, Fig. 6B). The presence of the other half of the M notes from auditory inputs significantly increased meter perception by 20% for triple (t = 2.39, p<.05) and 23% for duple perception (t = 3.52, p<.01) (white hashed bars, Tac C1/Aud C2, Fig. 6A and 6B). However, the presence of the other half of the M notes from tactile modality did not significantly change subjects’ performance (white hashed bars, Aud C1/Tac C2., Fig. 6A and 6B). These results support the results showing that the contribution of metric cues in meter perception is asymmetric, with audition playing a bigger role than touch.

When amplitude accents were added, performance increased 33% for unimodal auditory condition (t = 6.27, p<.01) and 20% for bimodal Aud-C1/Tac-C2 condition (t = 2.43, p<.05) for triple sequences, respectively. However, little change was observed for the unimodal tactile condition (gray bars, Fig. 6A) and bimodal Tac-C1/Aud-C2 condition (gray hashed bars, Fig. 6A). Similar results were observed for duple sequences (Fig. 6B).

Accented metric cues significantly enhanced the discriminability of meter when channel 1 was auditory input (Fig. 6C). Auditory input significantly enhanced the discriminability of meter when channel 1 was from tactile input for both accented and unaccented conditions (Fig. 6D). The results of Experiment 3 show that the roles of auditory and tactile stimulation in meter perception are asymmetric with auditory inputs having a larger effect.

### Experiment 4: Interference between Auditory and Tactile Channels in Bimodal Meter Perception

#### Test condition

In Experiment 4 (Fig. 3, Exp. 4), we tested how subjects deal with consistent or conflicting meter cues when simultaneously receiving auditory and tactile input. Congruent and incongruent tasks were tested in this experiment. In the congruent task, a note sequence was delivered through one channel and the same M notes were presented from the other channel. In the incongruent task, a note sequence was delivered through one channel and different M notes were presented to the other channel. Experiment 4 had eight test conditions as listed in Table 1. Each condition was tested in 16 trials, resulting in a total of 160 test trials.

#### Results for experiment 4

**Figure 7:**
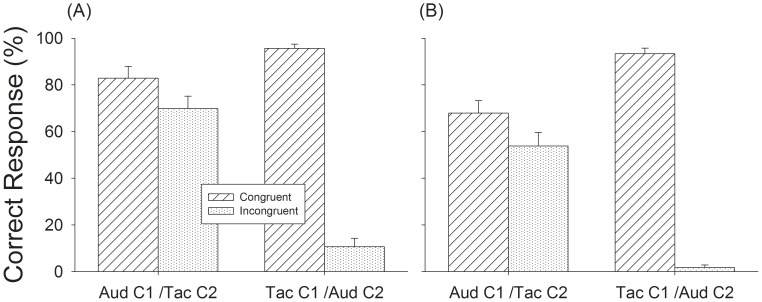
Results of Experiment 4, meter recognition performance in triple sequences (A) and duple sequences (B). Hashed bars show congruent condition and dotted bars show incongruent condition. Error bars are standard error.

In this experiment, we hypothesized that if the inputs from touch and audition are grouped then performance should be greatly affected when the two modalities provide conflicting input. Do the inputs sum or are they perceived separately? We examined the interaction between the two channels when the cues from the second channel were either congruent or incongruent to channel 1 (Fig. 3). In the congruent task, when the meter cues were the same for both channels, performance was high, with correct response to triple sequences at 83% and 96% for Aud-C1/Tac-C2 and Tac-C1/Aud-C2 conditions, respectively (Fig. 7A), and 68% and 93% for duple sequences (Fig. 7B). In the incongruent task, performance in triple sequences dropped to 70% and 11% and 54% and 2% for the duple sequences (Fig. 7B).

The effect of congruency was also measured by comparing performance in the bimodal conditions of Experiment 4 to the unimodal conditions of Experiment 1. Paired *t-test* showed that congruent auditory M notes significantly enhanced performance for tactile triple (t = 3.35, p<.01) (Fig. 4A Tac, vs. Fig. 7A Tac-C1/Aud-C2.) and duple (t = 5.36, p<.01) sequences. Congruent tactile M notes did not change auditory performance for triple sequences (t = −1.53, p>.05) (Fig. 4A Aud, vs. Fig. 7A Aud-C1/Tac-C2), and actually reduced performance for duple sequences (t = −6.00, p<.01). Repeated measures ANOVA indicated that the main effect of incongruent M notes on meter perception was significant [F_(1,12)_ = 86.38, p<.001] compared to unimodal tests (Experiment 1). Incongruent tactile M notes significantly decreased auditory performance for triple sequences by 20% (t = 3.32, p<.01) (Fig. 4A Aud vs. Fig. 7A Aud-C1/Tac-C2), and by 30% for duple sequences (t = 3.35, p<.01). Incongruent auditory M notes significantly decreased tactile performance by 72% for triple sequences (t = 10.03, p<.01) (Fig. 4A Tac, vs. Fig. 7A Tac-C1/Aud-C2) and 68% for duple sequences (t = 13.51, p<.01). These results support the notion that the interaction between auditory and tactile stimulation in meter perception is substantial, and confirms our previous observation that audition has a greater influence on meter perception than touch.

## Discussion

Music is generally considered to be an auditory experience. However, it is often accompanied by other sensory stimuli (e.g., proprioceptive, vestibular, tactile) and motor actions. When listening or playing music, one of the most prominent sensory inputs accompany audition is the sense of touch. A large number of tactile receptors innervate the skin and tissues of the body. In particular, the Pacinian afferents which are found in the skin and in large numbers in the omentum of the gut is exquisitely sensitive to minute vibrations as small as 100 angstroms [28]. In this study we hypothesized that inputs to these afferents could allow musicians to “feel” the rhythm of music and can contribute to listeners tapping their hands and feet to the rhythm. Our working hypothesis is that the tactile component of meter perception comes from the activation of the cutaneous Pacinian afferents, which are most sensitive to 220 Hz vibrations, and have been shown to faithfully encode temporal patterns of transmitted vibrations with similar physical qualities and temporal patterns as the sounds [28]. In this study we show that musical meter can be perceived through the activation of cutaneous mechanoreceptive afferents and further that meter information from audition and touch is grouped into a common percept and is not processed along distinct separate sensory pathways.

The testing sequences we used were similar to sequences that were used in previous studies of meter perception. Briefly, those studies showed that 1) humans can tap in synchrony with beats to the sequence of pure tactile hand stimulations [11]; 2) meter cannot be perceived through visual stimulation [15], [17]; 3) meter perception is influenced by interactions with the motor and vestibular systems [12]–[16], [18]–[21] and even 7-months-old infants have the ability to discriminate meter, suggesting that it is not a learned mechanism [7].

In the present study using young adults some musical training, we first showed that under unimodal conditions, subjects can perceive the implied meter patterns from auditory or tactile sequences with ambiguous rhythms (unaccented condition) at an average accuracy rate of about 82% (auditory) and 75% (tactile), respectively (Fig. 4A). This performance is slightly better than what Hannon et al [22] found in their auditory studies, which could be explained by our subjects having musical training and being older than those tested in the Hannon et al studies [7], [22], [23]. We then showed that unimodal tactile meter perception behaves like auditory meter perception that performance increases significantly when accent cues are added to key metrical notes (Fig. 4A). These results demonstrate that meter can be perceived through passive touch and further suggest that the mechanisms underlying tactile and auditory meter perception share similar characteristics.

In the next set of experiments we tested the degree that auditory and tactile inputs are grouped in processing meter. If, for example, the sensory systems process information independently, then presenting the inputs bimodally should not affect meter perception. We find that performance rose from chance when there are no meter cues to 70–90% with bimodal input (Fig. 5A). It should be stressed that subjects performed all of the experiments without training, feedback or instructions about where to focus their attention, demonstrating that auditory-tactile integration for meter perception is an automatic process. The results demonstrate, we believe, for the first time that auditory and tactile input are grouped during meter perception. Previous studies have failed to find sensory grouping across any sensory modalities (for review, see Spence and Chen, 2011) [43].

We further examined the asymmetry between auditory and tactile inputs in meter perception to address the modality dependent characteristics of meter perception. We explored whether auditory or tactile dominance by altering the balance between the relative strength of the auditory and tactile inputs, e.g. metrically important notes and accents. We found that the presence of metrically important notes from audition has a significantly larger influence on meter perception than when they are presented tactually, indicating that audition plays a dominant role in meter perception (Fig. 6). This dominance could be due to the level of stimulation that we used in the current study. Since the inputs to the auditory system came from head phones, the stimuli engaged a large number of receptors in the cochlea whereas only a tiny portion of tactile receptors (in this case, afferents innervating the left index finger tip) was used to process the tactile sequences. It is not clear if the dominance of audition over touch would persist if a larger area of the body were activated by tactile input (e.g., like during a loud rock concert). Although subjects reported subjectively that the perceived intensities of the stimuli were subjectively similar, intensity cues cannot be ruled out as playing a role given different stimulus conditions. The differences in how the two modalities are engaged should be considered when evaluating the dominance of one system over the other in meter perception and should be considered when evaluating studies showing that auditory inputs tend to dominate for the processing of rhythmic temporal stimuli [24]. These differences could explain why other studies have shown that audition appears to be minimally susceptible to the influences from other sensory inputs when perceiving temporal events [25].

In the last set of experiments we tested whether meter is processed along separate or common pathways. We found that while congruent stimulation enhanced meter perception, incongruent meter cues inhibited meter perception (Fig. 7). Again, we found that the integration between audition and touch was asymmetrical with auditory cues being weighted more strongly than tactile cues. One hypothesis is that when presented with conflicting input the conflict is resolved by suppressing one input in favor of another in a manner similar to the way that cross-modal sensory inputs tend to be captured by the modality that is most appropriate to the specific task. Another possibility is that attentional capture may play a role by suppressing irrelevant stimuli and subjects may have subconsciously directed their attention to the auditory input.

The neural mechanisms of meter perception are not well understood. There are many similarities shared by auditory and tactile systems that might contribute to metrical cue integration. Physically, auditory and tactile stimuli in these experiments are mechanical vibrations. In this study we used 220 Hz vibratory stimuli, which is the optimal range for activating the Pacinian afferents. The Pacinian afferent system has been proposed as being critical for processing tactile temporal input and plays an important role for encoding vibratory inputs necessary for tool use [26], [27]. The tactile inputs also activate the low frequency rapidly adapting afferents (RA) which are important for coding flutter [28]. There is evidence that the processing of low frequencies may be similar in audition and touch [29] and as such inputs from the RA afferents cannot be ruled out at this time.

Based on our results, we suggest that the brain treats the stimulus sequences from the two channels as one stream rather than as two independent streams. How the tactile inputs interact with auditory inputs is not understood. One possibility is that the integration is simply due to energy summation, but this does not explain the asymmetrical effects observed between the auditory and tactile inputs that we found in Exp. 3 and 4. The more likely explanation is that meter is processed along a common central neural pathway that receives inputs from both systems that modulated by inputs from the two systems with inputs from the auditory system inputs are weighted stronger than tactile inputs.

The interaction between hearing and touch in signal detection, frequency discrimination, and in producing sensory illusions are well documented (for a review, see [30]), and several studies report that there are central connections linking the auditory and tactile systems [31]–[36]. Candidate areas where the integration could take place are the cerebellum [37], premotor cortices, auditory cortex [38] as well as the superior prefrontal cortex [18]–[21]. Studies have suggested that auditory cortex is involved in tactile temporal processing and auditory rhythm perception activates dorsal prefrontal cortex, cerebellum and basal ganglia [21], [39]. A recent electroencephalogram study has suggested that a neural network that spans multiple areas, instead of specific brain area, may be the bases of beat and musical meter perception [40]. Although we show here the interaction between touch and audition, we speculate that multi-sensory cross-modal grouping of musical meter probably involves the integration of multiple sensory systems and could underlie the rhythmic movements associated with dance.

## Supporting Information

Figure S1 Results of Experiment 2, meter recognition in duple sequences for bimodal M/N split and M/N half split tasks. (A) duple sequences tested in the M/N split task, (B) duple sequences tested in the M/N half-split task. Open bars are results tested under unimodal condition. Hashed bars are results tested under bimodal condition. Error bars are standard error.
(TIF)
