# Supplementary figures and images for: Feeling Music: Integration of Auditory and Tactile Inputs in Musical Meter Perception
Source: PLoS One. 2012 Oct 31;7(10):e48496. doi: 10.1371/journal.pone.0048496 (PMC3485368; doi:10.1371/journal.pone.0048496)

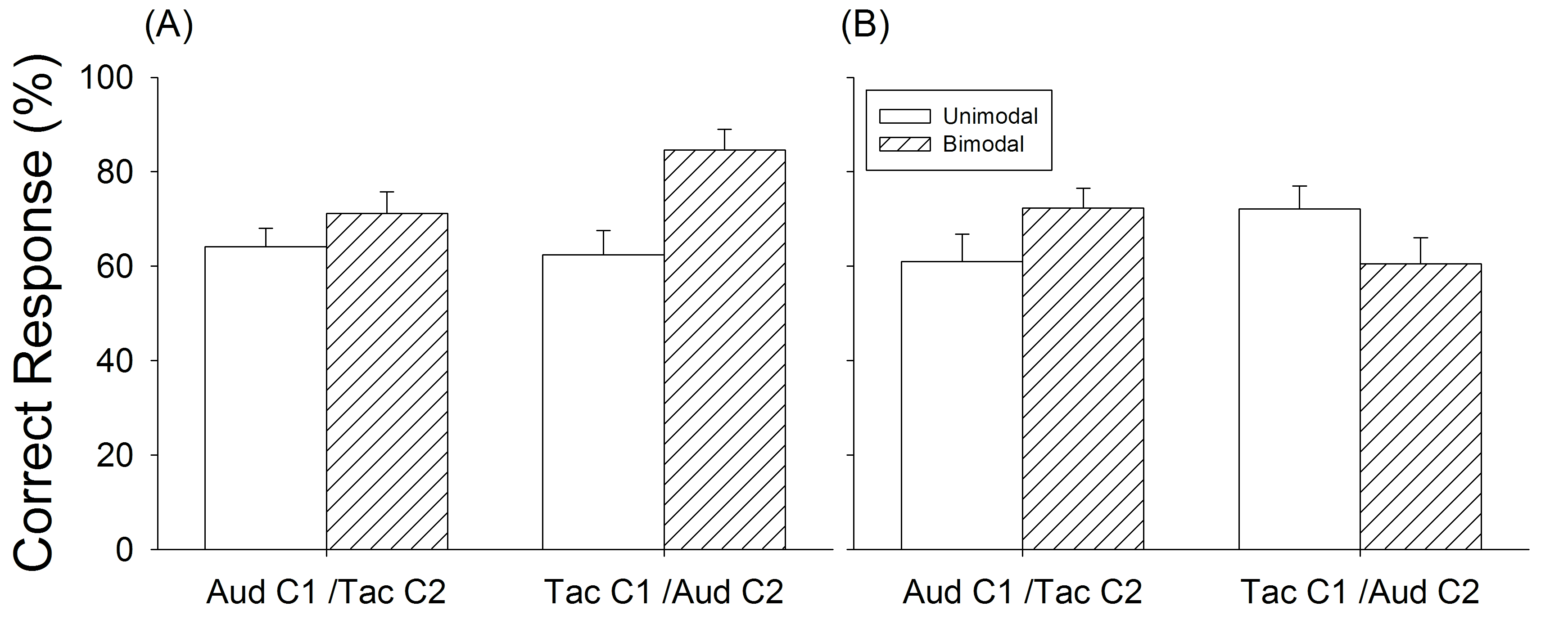

Supplement: Figure S1 — Results of Experiment 2, meter recognition in duple sequences for bimodal M/N split and M/N half split tasks. (A) duple sequences tested in the M/N split task, (B) duple sequences tested in the M/N half-split task. Open bars are results tested under unimodal condition. Hashed bars are results tested under bimodal condition. Error bars are standard error. (TIF) [file pone.0048496.s001.tif]
